# Wound Dressing: Iodoform Intoxication

**Published:** 1885-04-20

**Authors:** P. J. Hayes

**Affiliations:** F. R. C. S. Ed., Surgeon to the Mater Misericordiæ Hospital, &c., Dublin


### WOUND DRESSING: IODOFORM INTOXICATION.

By P. J. Hayes, F. R. C. S. Ed.,

**Surgeon to the Mater Misericordiæ Hospital, &c., Dublin.**

Recent experiences go to prove that the antiseptic properties of iodoform are not so available as to render it capable of becoming a substitute for cabolic acid. Morover, it requires to be employed with caution, as too free an application may induce fatal consequences. It will be as advisable to consider, in turn, the methods in vogue with surgeons who apply iodoform to wounds, the advantages which can be claimed for it as an antiseptic dressing, the dangers which are apt to follow its injudicious application, and the precautions to be observed when the employment of it appears advisable to the surgeon.

When the fancy for using iodoform, as a substitute for Listerism, seized the German surgeons, very large quantities of iodoform were oftentimes applied to extensive operation wounds—indeed so much as from three to six ounces was not considered to be an undue amount for introduction at a first dressing. Mostig-Moorhof, however, employs not more than from five to ten drachms. The ordinary method of using it is to shake it freely over the wounded surface, and to fill every cavity or offset from the wound with it; then to apply dressings of either iodoform gauze or boric cotton. Prof. Tripier recommends it to be applied in a more guarded manner than that by which so many unfortunate results have been caused in Germany. He always irrigates a wound with carbolic solution; and when there is so great a loss of substance that the wound-edges cannot be brought into contact, he lightly dusts the raw surface with iodoform, covering the cavity with Lister's gauze, When changing dressings he takes care not to disturb the previously applied iodoform, but after gentle washing he adds just enough of fresh iodoform to re-cover any exposed surface in the wound. This combined form of dressing he believes to be particularly suitable after operations about the natural orifices, in cases of contused or lacerated wounds of the hand, and where caseous glands have been cleared out with a sharp spoon. Tripier also favours the application of Moleschott's collodion (consisting of iodoform, 1 part; collodion, 15 parts) for incised wounds about the face. I think many surgeons in this country have had an experience as extensive and satisfactory of iodoform dressings as has been that of Prof. Tripier. The advantages which may fairly be claimed for iodoform are—that it helps to allay painful sensibility, and serves to diminish the quantity and alter the character of discharge (removing fœtor, and causing a purulent secretion to assume a more or less serous condition); its presence causes the subsidence of fungoid outgrowths, on the one hand, whilst on the other, it hastens the growth of healthy granulation-tissue; and, if it be not disturbed, is apt, with serous discharge, to form such a protective covering for the wound that healing, as under a scab, is likely to occur.

The ill effects of iodoform may be classed into those of minor degree and the veritable intoxication. Kænig of Gottingen, Zeissel, and others, have reported cases where an erythematous eruption followed the employment of iodoform—disappearing when it was withheld, and recurring after its application.

With regard to real poisoning or intoxication, the statistics of Kænig extended over 32 cases—19 men and 13 women—the majority of serious and fatal cases occuring amongst patients beyond the age of fifty years.

Kænig distinguishes two principal forms of intoxication—the light form and the grave. In the light form the patients experience anorexia, headache, loss of memory; they appear morose, or even delirious, cry without reason, and seem fearful of being persecuted.

The pulse becomes enormously accelerated—the temperature perhaps slightly elevated. Symptoms subside within a few days, recovery taking place. In the grave form hallucinations of variable nature occur; sometimes delirium assumes a furious character, or the patient becomes terror-stricken, and may even exhibit a disposition to commit suicide. Food is refused; urinary secretion becomes scanty; the pulse is rapid; the temperature rises to perhaps 104°F.; the *denouement* is almost always fatal, the patient dying in a state of coma.

Young subjects generally present symptoms resembling those of meningitis, but usually without elevation of temperature.

Kænig and Aschenbrandt have, like Martin, observed, at post mortem examinations, evidences of iodoform-pneumonia.

I think few surgeons will be found to oppose the recommendations of Martin and Velaminoff, that iodoform should, in all cases where its use is indicated, be applied in small quantity; that especial caution should attend its use when much adipose tissue is exposed, and when the wound is extensive; that its employment is more or less contra-indicated by an advanced age, tendency to fatty degenerations, diseased conditions of heart or lungs, and when there exists a susceptible condition of the nerve centres.—*Dublin Medical Journal, August, 1883, p. 110.*

